# Supplementary figures and images for: The Regulatory Role of Myomaker in the Muscle Growth of the Chinese Perch (Siniperca chuatsi)
Source: Animals (Basel). 2024 Aug 23;14(17):2448. doi: 10.3390/ani14172448 (PMC11394465; doi:10.3390/ani14172448)

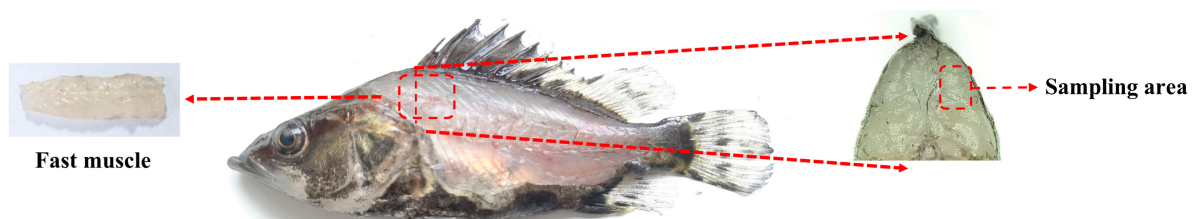

**Figure S1.** Schematic diagram of fast-muscle sampling of Chinese perch.

Supplement: Supplementary file 1 [file animals-14-02448-s001.zip › animals-3139272-supplementary.pdf]
